# Triple-negative ectopic breast cancer of the male scrotum: a case report

**DOI:** 10.3389/fonc.2024.1473881

**Published:** 2024-12-05

**Authors:** Yuhui Nie, Chen Song, Jingru Wang, Kun Wu, Mingxin Yu, Xin Shen, Yandong Bian, Shuzhen Liu

**Affiliations:** ^1^ School of Clinical Medicine, Shandong Second Medical University, Weifang, Shandong, China; ^2^ Weifang People’s Hospital, Shandong Second Medical University, Weifang, Shandong, China; ^3^ Center for Precision Pathological Diagnosis, Weifang People’s Hospital, Shandong Second Medical University, Weifang, Shandong, China

**Keywords:** scrotal mass, ectopic breast cancer, triple negative, Paget’s disease, treatments

## Abstract

Male breast cancer represents only 1% of all breast malignancies, with ectopic breast cancer in men being even rarer and highly prone to diagnostic challenges. Extramammary Paget’s disease (EMPD), a rare cutaneous tumor with non-specific clinical symptoms, is susceptible to misdiagnosis. This report discusses the case of an older male patient who presented with a scrotal mass, later identified as ectopic breast invasive adenocarcinoma upon pathological examination post-lesion excision. Immunohistochemistry confirmed a triple-negative profile and EMPD diagnosis, with no malignancies detected in either breast. Despite multiple treatment regimens and recurrence following adjuvant chemotherapy, the disease progressed with associated chemotherapy-related side effects, resulting in a 25.5-month survival period. The scarcity of literature on male ectopic breast cancer complicates the understanding of its incidence and optimal treatment strategies, increasing the risk of misdiagnosis. This study highlights the diagnostic and therapeutic challenges of this rare case, emphasizing the need for early recognition of atypical manifestations. The manuscript aims to assist clinicians by sharing case-specific insights and reviewing pertinent literature to enhance comprehension and management of similarly rare cases.

## Introduction

Embryonic mammary development begins around the fourth week of gestation, forming a ventral mammary ridge extending from the axilla to the inner thigh. Incomplete resorption of this tissue can lead to residual ectopic mammary glands (1). Although ectopic breast tissue undergoes similar pathophysiological changes as normal breast tissue, only an insignificant fraction (approximately 1%) develops cancer in these sites (2). Ectopic mammary glands are most commonly identified in the axillae of women, with cases in men, particularly in the scrotal region, being significantly rare and cancer in these sites even rarer. Paget’s disease (PD), initially described by James Paget in the breast in 1874, was later identified in the male genital region by Crocker in 1889. This condition primarily involves intraepidermal adenocarcinoma, characterized by malignant growth of non-keratinizing epithelial cells known as Paget cells. Scrotal involvement in extramammary PD (EMPD) is uncommon, accounting for only 14% of cases, compared to the vulvar type (65%) and perianal type (20%) (3). This study details a rare case of breast cancer originating in ectopic mammary tissue within the scrotum of a male patient, accompanied by EMPD of the scrotal skin, highlighting the diagnostic and therapeutic challenges posed by this uncommon condition.

## Case report

**Figure 1:**
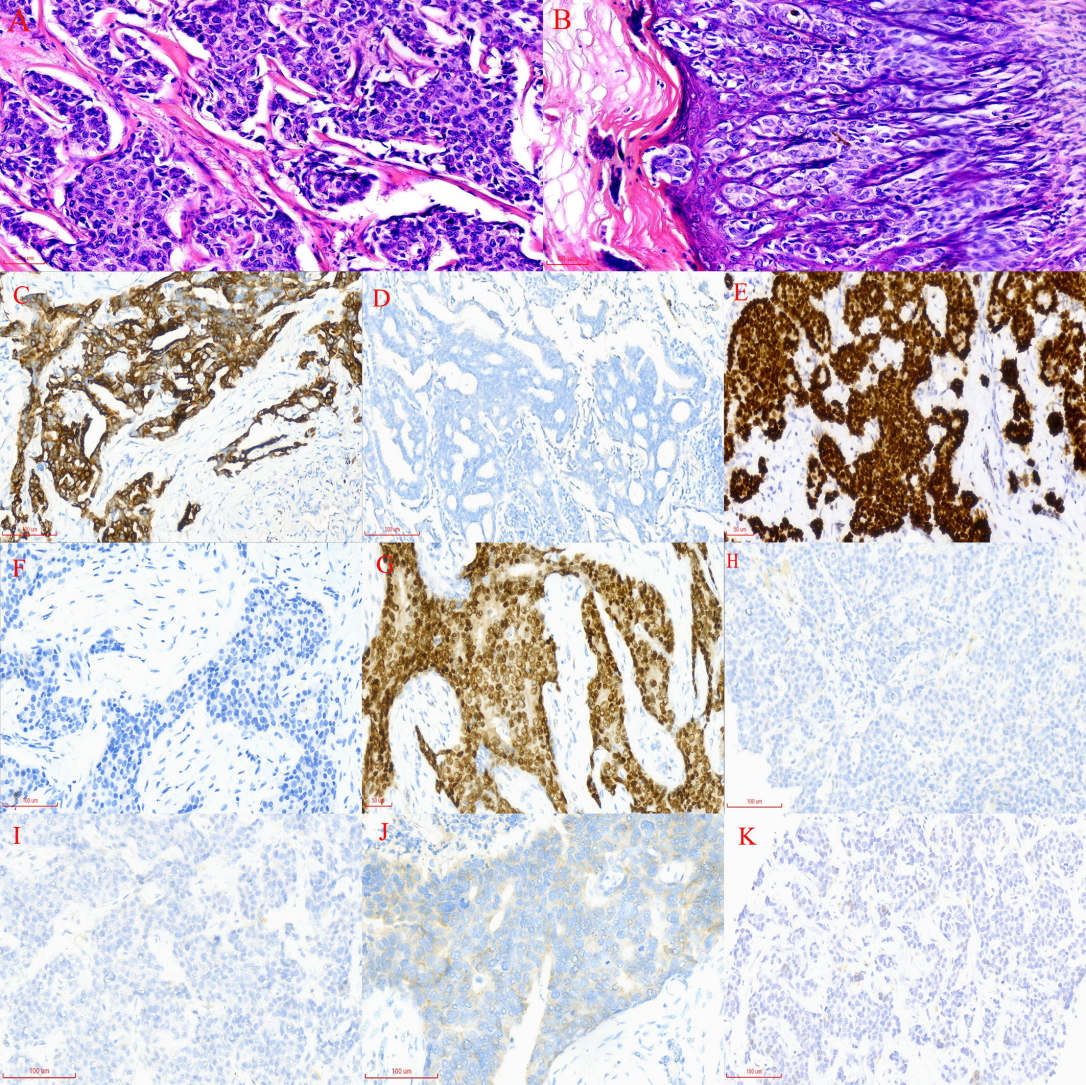
**(A)** Widespread infiltration of cancer cells in the scrotal subcutaneous tissue, with indistinct cellular borders, increased nuclear-cytoplasmic ratio, acidophilic cytoplasm, deviated nuclei, basophilic staining, enlarged nuclear volume, pronounced heterogeneity, and pathological nuclear division (magnification, x200). **(B)** Irregularly arranged epidermal cells with varying sizes, red-stained cytoplasm, and blue-stained nuclei, identified as Paget’s cells. These cells exhibit large nuclei with loose chromatin, prominent nucleoli, and irregular edges; they are distributed in a band-like pattern along the epidermis (magnification, x200). **(C)** CK-7 immunohistochemical staining of the subcutaneous scrotal mass: brownish-yellow staining indicates CK-7 positive cells, while hematoxylin-stained nuclei appear blue (×200). **(D)** CK-20 immunohistochemical staining of the subcutaneous scrotal mass: absence of brown staining indicates no CK-20 expression in the sample (×200). **(E)** GATA-3 immunohistochemical staining of the subcutaneous scrotal mass: brown staining highlights GATA-3 positive cells (×200). **(F)** GCDFP-15 immunohistochemical staining of the subcutaneous scrotal mass: lack of brown staining indicates no GCDFP-15 expression in the sample (×200). **(G)** AR immunohistochemical staining of the subcutaneous scrotal mass: brown staining denotes AR-positive cells, with approximately 60% of cells showing positivity (×200). **(H)** Immunohistochemical ER nuclear staining in scrotal dermal cells displaying a light blue color (magnification, x200). **(I)** Immunohistochemical PR nuclear staining in scrotal dermal cells displaying a light blue color (magnification, x200). **(J)** Immunohistochemical Human Epidermal Growth Factor Receptor 2 nuclear staining in scrotal dermal cells appearing pale blue (magnification, x200). **(K)** PD-L1 testing in liver metastatic tissue using the CPS, calculated by dividing the number of PD-L1 positive cells (including tumor cells, lymphocytes, and macrophages) by the total number of viable cells, then multiplying by 100. A CPS of <1 indicates low PD-L1 expression (magnification, x200).

**Figure 2:**
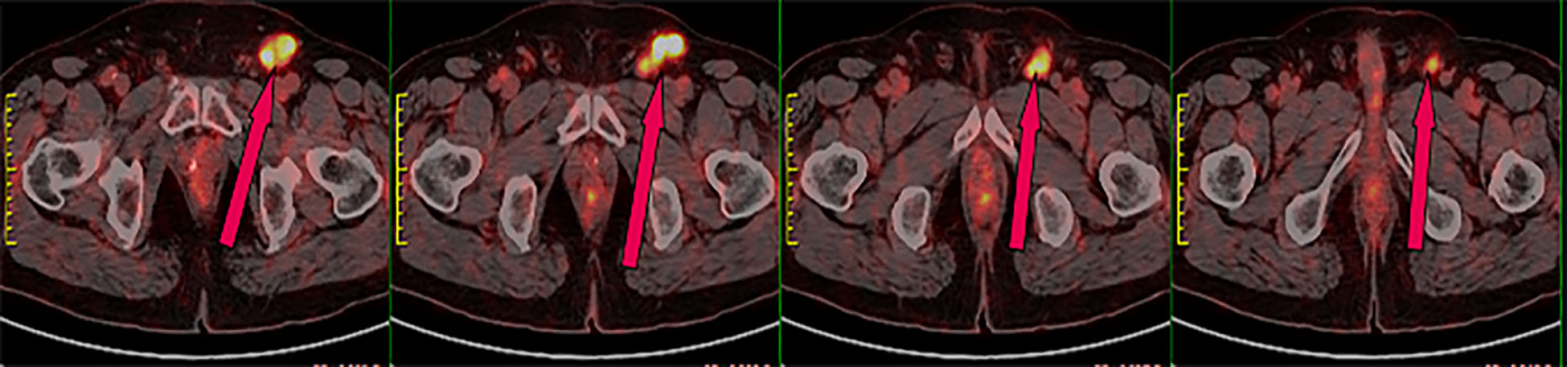
PET-CT scan showing radioactive uptake in the left inguinal lymph nodes, indicative of malignant metastasis.

**Figure 3:**
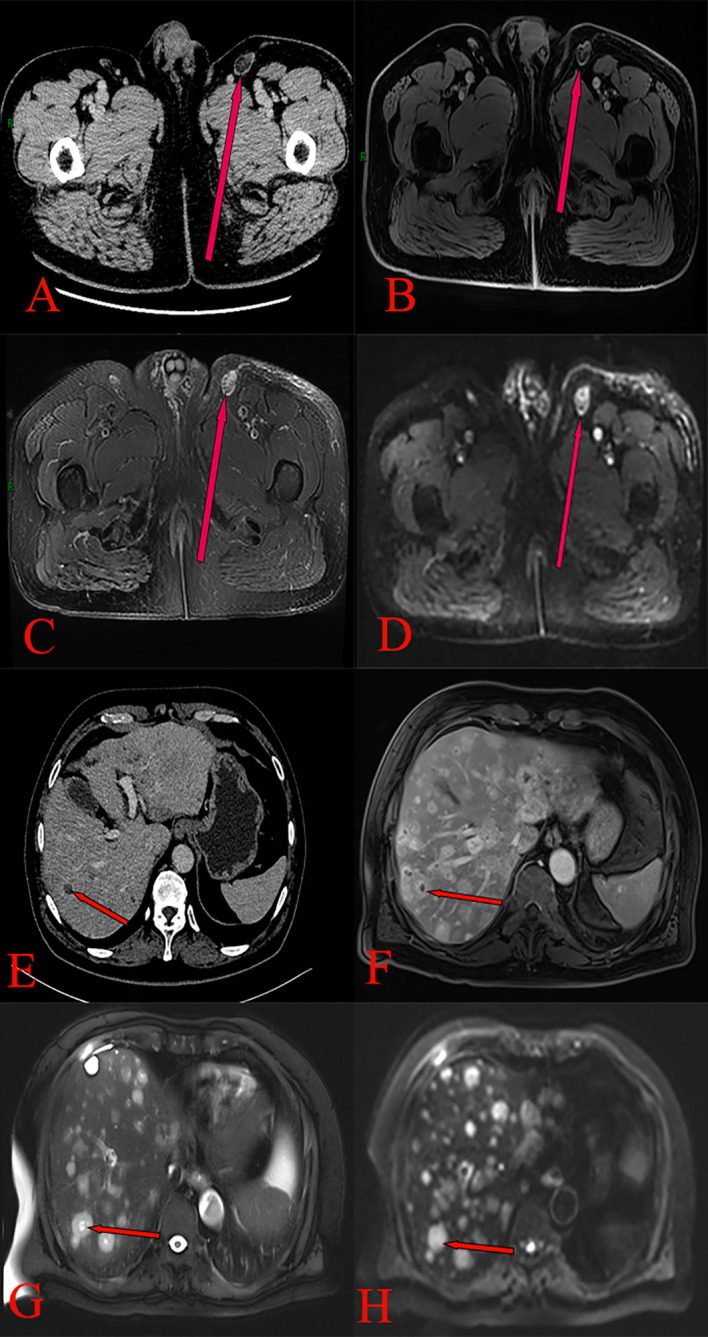
**(A)** Pelvic CT scan showing an irregular soft tissue density mass in the left inguinal region, with blurred borders and mild enhancement. **(B)** Pelvic MRI T1 phase pelvic image showing a low-signal nodular shadow in the left inguinal region. **(C)** Pelvic MRI T2 phase showing high-signal nodular shadows in the left inguinal region, with fat suppression resulting in a high signal. **(D)** Pelvic MRI DWI phase demonstrating restricted diffusion in a left inguinal nodule. **(E)** Enhanced CT scan of the upper abdomen showing multiple rounded hypodense liver lesions, exhibiting no enhancement. **(F)** Upper abdominal MRI T1 phase revealing multiple round, long T1 signal shadows in the liver. **(G)** Upper abdominal MRI T2 phase revealing multiple round, long T2 signal shadows in the liver, with substantial enhancement on the contrast-enhanced scan. **(H)** Upper abdominal MRI DWI phase revealing multiple intrahepatic round signal shadows with limited diffusion.

In June 2020, a 63-year-old male patient with no family history of malignancies presented to our hospital’s surgical department with a 5-month history of a scrotal skin lesion near the base of the penis, accompanied by occasional pain, no discharge, and ineffective self-medication attempts. The patient reported no discomfort in the axilla or breast, and physical examination revealed no palpable masses. Ultrasound imaging showed subcutaneous hypoechoic tissue at the base of the penis, measuring 1.3 cm × 0.7 cm × 1.7 cm, with regular morphology, indistinct borders, and a significant blood flow signal. Moreover, imaging of the kidneys, ureter, and bladder (including the prostate) showed no abnormalities. Due to limited awareness of the condition, a multidisciplinary surgical strategy discussion was not conducted, and a simple excision of the scrotal mass was performed. Postoperative pathological examination confirmed the scrotal mass as an invasive adenocarcinoma. (**Figure 1A**), with EMPD of the scrotal skin (**Figure 1B**), as well as evidence of nerve invasion and cancerous embolism in the chorioallantoic duct. The immunohistochemical analysis revealed cytokeratin 7 (CK7) positivity, CK20 negativity, GATA3 positivity, gross cystic disease fluid protein 15 (GCDFP-15) negativity, and raised androgen receptor (AR) (3+, 60%) (**Figures 1C–G**), with estrogen receptor (ER) and progesterone receptor (PR) negativity, and a human epidermal growth factor receptor 2 (C-erB-2) score of 2+ (**Figures 1H–J**). Further Fluorescence *In Situ* Hybridization (FISH) testing yielded negative results. Based on these pathological and immunohistochemical results, a diagnosis of triple-negative invasive carcinoma of ectopic breast origin was made. Following the surgical procedure, the patient consulted the Thyroid and Breast Surgery Department for further breast examination. A positron emission tomography (PET)-computed tomography (CT) scan detected multiple lymph node metastases in the left inguinal region (**Figure 2**), leading to an inguinal lymph node dissection. Postoperative pathology confirmed metastasis from the scrotal invasive adenocarcinoma. Two months later, pelvic CT and magnetic resonance imaging (MRI) identified a space-occupying lesion in the left inguinal region (**Figures 3A–D**), raising suspicion for inguinal lymph node metastasis. Systemic chemotherapy and regular monitoring were recommended.

**Figure 4:**
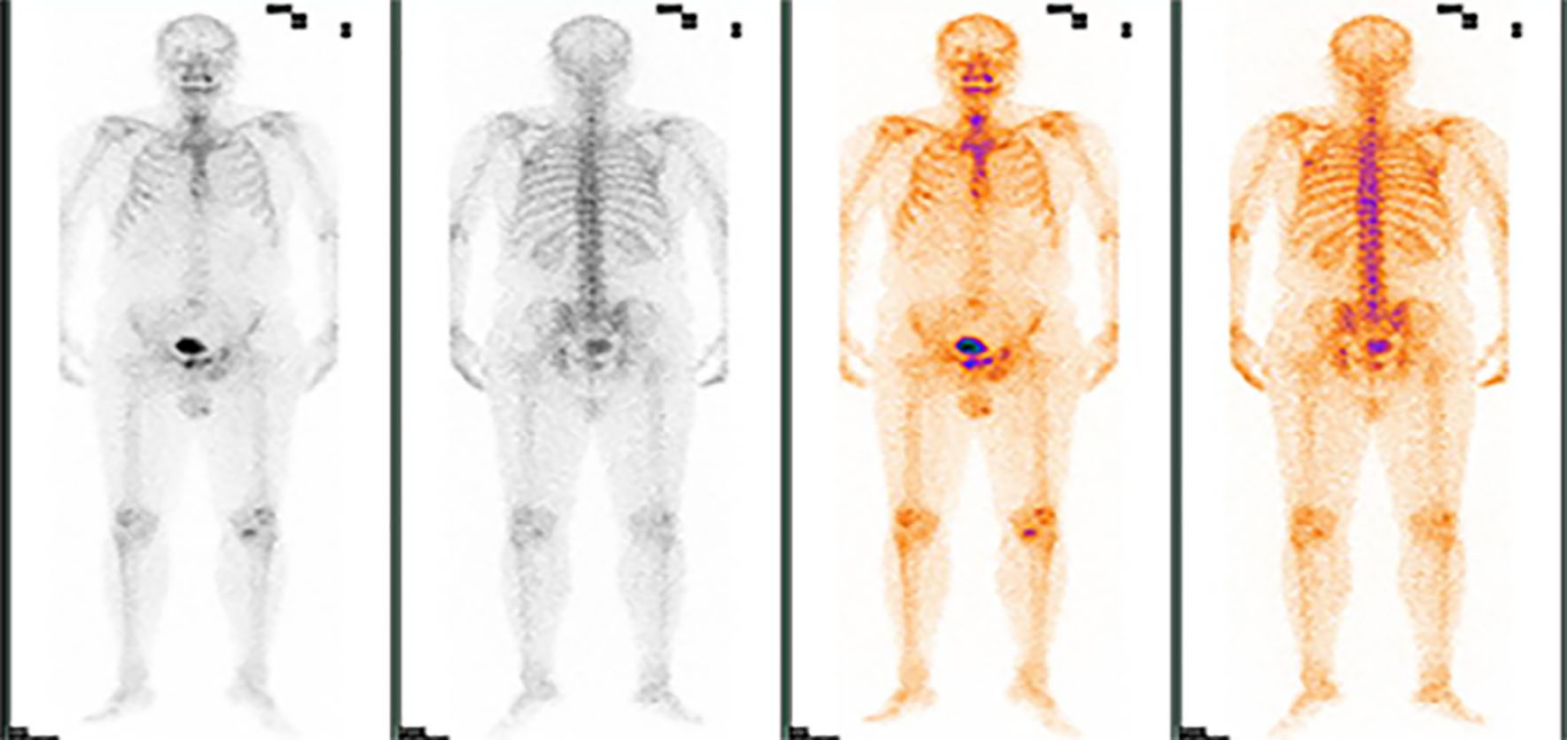
ECT demonstrating foci of abnormal radiological distribution with varying morphologies and sizes in the bilateral suprapubic branches, left acetabulum, and other regions.

Based on the PET-CT findings and prior inguinal lymph node dissection, an aspiration biopsy was not performed for the suspected lymph node metastasis noted on pelvic imaging. The patient subsequently received four cycles of adjuvant chemotherapy with paclitaxel liposomal (175 mg/m²) and nedaplatin (75 mg/m²) from August to October 2020, resulting in disease stability upon follow-up. In August 2021, the patient presented again with a scrotal mass, which was excised and confirmed to be an invasive adenocarcinoma of ectopic mammary origin. During postoperative re-examination, an ultrasound of the inguinal lymph nodes once more indicated metastasis, prompting the patient to return to our department for further treatment. Further investigations revealed metastatic spread to the liver (**Figures 3E–H**), bilateral pubic bones, and the left acetabular bone (**Figure 4**). A liver mass biopsy confirmed metastatic invasive adenocarcinoma with immunohistochemistry consistent with triple-negative status. Programmed death-ligand 1 (PD-L1) testing indicated a combined positive score (CPS) of less than 1, assessed on the Dako platform with the 22C3 antibody clone using the CPS standard (**Figure 1K**), suggesting ineligibility for immune checkpoint inhibitor therapy. The patient experienced rapid disease progression, with multiple pathological examinations indicating ectopic breast origin. A physical and ultrasound examination of the breast and axilla revealed no significant abnormalities. Given the patient and their family’s urgent desire for treatment, chemotherapy with the TE regimen (albumin-bound paclitaxel 250 mg/m² and epirubicin 70 mg/m²) was initiated following a multidisciplinary consultation. Although denosumab has demonstrated better efficacy and safety over zoledronic acid for bone metastasis management, due to constraints related to national insurance coverage and personal financial limitations, the patient opted for zoledronic acid, administered every three weeks, to manage bone metastasis while minimizing financial burden. During this period, the treatment achieved partial remission (PR). However, after seven cycles, treatment was discontinued due to secondary neurotoxic effects, including head and facial paresthesia, and extremity numbness. The patient was subsequently transitioned to maintenance chemotherapy with capecitabine (1250 mg/m^2^). After two cycles, intrahepatic metastases progressed, promoting a repeat biopsy and confirming triple-negative status via immunohistochemistry. Subsequently, the patient received a single cycle of gemcitabine (1 g/m^2^), carboplatin (area under the curve [AUC] = 5), and bevacizumab (7.5 mg/kg). However, side effects, including grade 4 thrombocytopenia and grade 3 leukopenia, necessitated platelet transfusions, administration of recombinant human thrombopoietin, and support to restore leukocytes and platelets. Consequently, gemcitabine was not administered on day 8 of this cycle.

Upon discharge, the patient was referred to a higher-level hospital for further pathological evaluation. The findings revealed metastatic adenocarcinoma in the left inguinal lymph nodes (2/4), consistent with scrotal invasive adenocarcinoma, and a liver puncture showed metastatic adenocarcinoma. Given these results, scrotal ectopic breast origin was considered. Subsequently, the patient received vedolizumab (120 mg) at the same hospital. During hospitalization, the patient developed a high fever reaching 39.7°C, initially treated with piperacillin-tazobactam followed by meropenem; however, the infection persisted with limited improvement. Despite intensive treatment efforts from the medical team and the patient’ family, therapeutic options were limited due to specific molecular profiles and expression levels of relevant markers.

A comprehensive timeline chart was developed to visually represent the patient’s treatment history and key clinical events (**Table 1**). This table systematically documents the patient’s journey from initial symptom recognition to final treatment, highlighting significant interventions such as operation, imaging, and chemotherapy. By providing a clear overview of the treatment process and its influence on disease progression, this timeline is a valuable reference to inform clinical decision-making.

**Table 1:** Comprehensive treatment timeline of the patient.

Date	Event	Category
January 2020	Patient discovered a scrotal mass near the base of the penis, red with occasional pain.	Initial Observation
June 6, 2020	Presented to Urology Department.	Initial Diagnosis
June 8, 2020	Scrotal lesion excision surgery performed under spinal anesthesia.	Surgery
June 12, 2020	Postoperative pathology showed invasive adenocarcinoma with Extramammary Paget's disease of the scrotal skin.	Pathology
June 28, 2020	PET/CT showed multiple hypermetabolic lymph nodes in the left inguinal region, indicating metastasis.	Imaging
July 8, 2020	Underwent inguinal lymph node dissection; multiple enlarged lymph nodes fused into a mass.	Surgery
August 6, 2020	Chest-abdominal-pelvic CT showed left inguinal mass.	Imaging
August 13 - October 25, 2020	Completed four cycles of chemotherapy with paclitaxel liposome and nedaplatin.	Chemotherapy
August 28, 2021	Follow-up revealed a recurrent scrotal mass.	Follow-up
September 6, 2021	Second scrotal lesion excision surgery performed.	Surgery
October 14, 2021 - March 6, 2022	Seven cycles of TE regimen chemotherapy completed.	Chemotherapy
April 20, 2022	Started capecitabine maintenance therapy.	Maintenance Therapy
May 11, 2022	Enhanced CT showed multiple liver metastases, indicating disease progression.	Imaging
May 13, 2022	Started treatment with gemcitabine, carboplatin, and bevacizumab; developed thrombocytopenia.	Chemotherapy
June 13, 2022	Received disitamab vedotin treatment; developed fever during hospitalization.	Treatment
July 2022	Patient passed away.	Outcome

PET/CT, Positron Emission Tomography/Computed Tomography; CT, Computed Tomography; TE, Nab-Paclitaxel + Epirubicin; EMPD, Extramammary Paget's disease.

## Discussion

Breast cancer is one of the most prevalent malignancies worldwide, predominantly affecting women and typically arising in the breast tissue. Ectopic breast cancer, a rare variant, develops along the embryonic milk line, which extends from the axilla to the groin, with the axilla being the most common site for ectopic breast cancer. To date, only three cases of male ectopic breast cancer outside the axilla have been documented in the English literature, with sites including the abdominal wall, perineum, and suprapubic region (4). In this report, a case of ectopic breast cancer is reported in the scrotum, situated within the perineal area. Furthermore, the patient was diagnosed with Paget’s disease, which added complexity to both the diagnostic and treatment approach (5).

Ectopic breast tissue is more susceptible to malignant transformation than normal breast tissue, primarily due to ductal stagnation. However, ectopic breast cancer remains uncommon, given the low incidence of ectopic breast tissue (6). The incidence of male breast cancer accounts for less than 1% of all breast cancers, and ectopic breast cancer represents approximately 0.3% to 0.6% of these cases (7, 8). The average age of diagnosis for ectopic breast cancer is approximately 54 years, roughly 6 years younger than the average age of diagnosis for conventional breast cancer (4). Ectopic breast cancer lacks distinct symptoms; common manifestations include palpable masses with or without tenderness, and skin changes such as erythema, ulcers, and other lesions (8, 9). However, due to the low incidence and its higher prevalence among women, there is limited diagnostic and treatment experience for male ectopic breast cancer, frequently resulting in delayed diagnosis. In the perineal region, EMPD can present with skin manifestations similar to those of ectopic breast cancer. Extramammary Paget’s disease is an adenocarcinoma originating in the skin or appendages, primarily affecting apocrine gland regions. Its main sites of occurrence are the vulva, followed by the perianal area, scrotum, penis, and axilla, predominantly affecting older individuals aged 60 to 70 years (10, 11). In a study of 246 Asian male patients with EMPD, the average age of onset was found to be 64 years (12). Research by Yin et al. indicates that the crude incidence rate of EMPD in mainland China is approximately 0.4 per million population (13). Besides being rare, EMPD presents with non-specific symptoms; initial manifestations commonly include itching, erythema, and dryness, which can progress to eczematous lesions, crusting, ulcers, or papillomatous changes. Therefore, patients may undergo prolonged treatments before a definitive diagnosis is made. Topical steroids or antifungal medications can further alter skin manifestations, complicating diagnostic processes (14).

Ectopic breast cancer and EMPD in the perineal region share similar clinical features, and the patient’s age of onset (63 years) aligns with the typical age range for EMPD, which can add to diagnostic challenges. Extramammary Paget’s disease is classified into primary and secondary types, with primary EMPD being CK7 positive and CK20 negative. Conversely, secondary EMPD is usually associated with an underlying malignancy and shows CK7 and CK20 positivity (15). In this case, the scrotal Paget’s disease is classified as primary EMPD, unrelated to ectopic breast cancer, with the scrotal lesions representing EMPD manifestations. The final diagnosis was ectopic breast cancer in the scrotum with coexisting EMPD.

Male ectopic breast cancer is sporadic, making the prognosis uncertain, and there is currently no established expert consensus on its management (16, 17). Treatment for male ectopic breast cancer generally follows protocols similar to those for primary breast cancer, primarily involving surgical excision, supported by chemotherapy, radiotherapy, endocrine therapy, targeted therapy, and the recently emerging immunotherapy. It is noteworthy that ectopic breast cancer exhibits greater aggressiveness than typical breast cancer, primarily in two aspects: first, the rate of lymph node positivity is higher than in breast cancer (18). Patients with vulvar ectopic breast cancer who underwent local wide excision and inguinal lymph node dissection showed pathological results indicating lymph node involvement in all cases (19). Second, ectopic breast cancer has a higher propensity for recurrence and metastasis, particularly to the bones and brain, following simple excision (20). Despite the increased likelihood of lymph node involvement, sentinel lymph node biopsy is not recommended for patients with ectopic breast cancer due to the reduced sensitivity of inguinal lymph nodes in dye uptake (21). To mitigate the risk of distant metastasis, postoperative local radiotherapy is recommended. Apart from traditional prognostic factors such as anatomical tumor-node-metastasis (TNM) staging, molecular subtype, histological grade, and Ki-67 index (22), genetic factors should be incorporated into traditional prognostic models, which could enhance prediction accuracy for male ectopic breast cancer. Currently, tests such as Oncotype DX (Exact Sciences Corporation, Madison, Wisconsin, USA) and MammaPrint (Agendia N.V., Amsterdam, The Netherlands) have shown utility in assessing the likelihood of distant metastasis (23, 24).

However, EMPD is relatively less invasive, with slow disease progression (17, 20). Surgical treatment remains the primary approach for EMPD, supplemented by options including laser ablation, radiotherapy, chemotherapy, and topical treatment comprising 5% imiquimod cream or cytotoxic agents combined with 1% fluorouracil cream (25, 26).

The patient’s prognosis was poor, with an overall survival of only 25.5 months. Critical contributing factors include the tumor’s triple-negative molecular subtype, which has limited treatment options; poor responsiveness to treatment, with rapid recurrence and metastasis following chemotherapy despite multiple treatment lines; and the rarity of the disease, which led to delayed diagnosis, limited understanding of its characteristics, and an initial treatment plan that was not optimally tailored to the patient’s specific needs.

Scrotal ectopic breast cancer and EMPD present with atypical symptoms and lack specific imaging findings, highlighting the importance of preoperative multidisciplinary consultation. Such consultation is essential for defining the surgical strategy, postoperative adjuvant treatment plan, and follow-up protocol. In this case, the patient did not undergo multidisciplinary evaluation, and only a simple lesion excision was performed, resulting in a non-standardized treatment course that led to recurrence and distant metastasis. Based on our experience, to minimize the risk of local recurrence, metastasis, or the malignant transformation of residual ectopic breast tissue, all cases of ectopic breast cancer are recommended to undergo evaluation by a multidisciplinary team (including surgeons, oncologists, radiation oncologists, radiologists, and pathologists) to address critical questions:

1. What should the surgical strategy be? Should a local excision or extensive resection be performed? Is sentinel lymph node biopsy necessary, or should lymph node dissection be considered?
2. What is the optimal adjuvant treatment? Given the rarity of ectopic breast cancer, treatment plans should be personalized, taking into account the patient’s risk factors, tumor characteristics, and overall health.
3. What is the best follow-up plan? What should be the frequency of follow-up visits? Which diagnostic tests should be included in the follow-up regimen?

## Conclusion

Scrotal ectopic breast cancer and scrotal Paget’s disease are exceedingly uncommon conditions characterized by non-specific early symptoms and the lack of distinctive findings on imaging. Diagnosis relies entirely on histopathological examination. Therefore, when conventional treatments for skin lesions prove ineffective, a high index of suspicion should be maintained, and a biopsy should be performed. Once a diagnosis is confirmed, a multidisciplinary expert consultation is essential to create a personalized treatment plan tailored to the patient’s unique case.

## Limitations

This study has several limitations. First, it is based on a single case, which limits the generalizability and extrapolation of the results. Second, the PD-L1 testing used the 22C3 diagnostic reagent. However, current research suggests that in triple-negative breast cancer, 22C3 and SP142 cannot be used interchangeably. Due to the limitations of the hospital platform, only 22C3 was available. Both reagents should ideally be tested simultaneously to ensure patients are not excluded from immunotherapy. Finally, after bone metastasis developed, zoledronic acid was chosen for treatment due to national insurance coverage and the patient’s financial constraints. Without significant economic pressure, denosumab, which is more effective with fewer side effects, should be the preferred treatment for bone metastasis.

## Data Availability

The original contributions presented in the study are included in the article/supplementary material. Further inquiries can be directed to the corresponding author.
